# Review of molecular subtyping methodologies used to investigate outbreaks due to multidrug-resistant enteric bacterial pathogens in sub-Saharan Africa

**DOI:** 10.4102/ajlm.v8i1.760

**Published:** 2019-03-14

**Authors:** Anthony M. Smith

**Affiliations:** 1 Centre for Enteric Diseases, National Institute for Communicable Diseases, National Health Laboratory Service, Johannesburg, South Africa; 2 Faculty of Health Sciences, University of the Witwatersrand, Johannesburg, South Africa

## Abstract

**Background:**

In sub-Saharan Africa, molecular epidemiological investigation of outbreaks caused by antimicrobial-resistant enteric bacterial pathogens have mostly been described for *Salmonella* species, *Vibrio cholerae, Shigella* species and *Escherichia coli*. For these organisms, I reviewed all publications describing the use of molecular subtyping methodologies to investigate outbreaks caused by multidrug-resistant (MDR) enteric bacterial infections.

**Objectives:**

To describe the use of molecular subtyping methodologies to investigate outbreaks caused by MDR enteric bacterial pathogens in sub-Saharan Africa and to describe the current status of molecular subtyping capabilities in the region.

**Methods:**

A PubMed database literature search (English language only) was performed using the search strings: ‘Africa outbreak MDR’, ‘Africa outbreak multi’, ‘Africa outbreak multidrug’, ‘Africa outbreak multi drug’, ‘Africa outbreak resistance’, ‘Africa outbreak resistant’, ‘Africa outbreak drug’, ‘Africa outbreak antibiotic’, ‘Africa outbreak antimicrobial’. These search strings were used in combination with genus and species names of the organisms listed above. All results were included in the review.

**Results:**

The year 1991 saw one of the first reports describing the use of molecular subtyping methodologies in sub-Saharan Africa; this included the use of plasmid profiling to characterise *Salmonella* Enteritidis. To date, several methodologies have been used; pulsed-field gel electrophoresis analysis and multilocus sequence typing have been the most commonly used methodologies. Investigations have particularly highlighted the emergence and spread of MDR clones; these include *Salmonella* Typhi H58 and *Salmonella* Typhimurium ST313 clones. In recent times, whole-genome sequencing (WGS) analysis approaches have increasingly been used.

**Conclusion:**

Traditional molecular subtyping methodologies are still commonly used and still have their place in investigations; however, WGS approaches have increasingly been used and are slowly gaining a stronghold. African laboratories need to start adapting their molecular surveillance methodologies to include WGS, as it is foreseen that WGS analysis will eventually replace all traditional methodologies.

## Introduction

For molecular epidemiological investigation of outbreaks in sub-Saharan Africa caused by antimicrobial-resistant (including multidrug-resistant [MDR]) enteric bacterial pathogens, most published data describe pathogens belonging to the genus or species of *Salmonella, Vibrio cholerae, Shigella* and *Escherichia coli*. By ‘molecular epidemiological investigation’, I refer to the use of molecular subtyping techniques that analyse bacterial strains at the level of their nucleic acid and so gives an indication of genetic similarity of strains.^1^ Molecular subtyping is vital for accurate epidemiological investigations of bacterial infections.^2^ Molecular subtyping allows one to segregate unlike strains of the same species or serotype of bacteria and identify clones or clusters of bacteria (genetically related strains). Molecular subtyping allows one to track the spread of strains or clones and determine how local strains compare to those circulating worldwide.^3^ Molecular subtyping data is critical for successful epidemiological investigation of outbreaks of disease; in particular, outbreak-related cases of disease can be differentiated from sporadic cases of disease.^4^ Knowledge of the molecular epidemiology of bacterial infections provides information of major circulating (infecting) clones, so that in times of antimicrobial treatment and vaccine interventions, well-informed and educated decisions can be made to combat the disease.^5^

**FIGURE 1:**
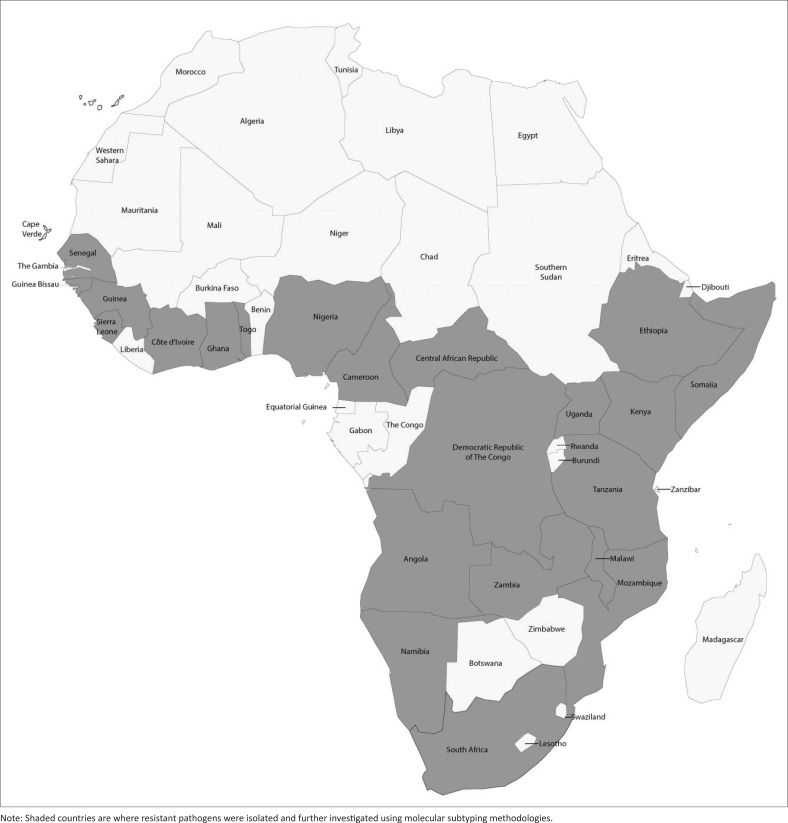
Map of Africa highlighting (with shading) countries where antimicrobial-resistant enteric bacterial pathogens have been isolated and further investigated using molecular subtyping methodologies.

The mid-1990s saw the start of noteworthy publications describing the use of molecular subtyping techniques to investigate the molecular epidemiology of antimicrobial-resistant enteric bacterial pathogens in sub-Saharan Africa.^6^ These molecular subtyping techniques included plasmid profiling, ribotyping (southern blotting of restricted genomic DNA and probing with ribosomal genes), random amplified polymorphic DNA analysis (also called arbitrarily primed polymerase chain reaction [PCR]), enterobacterial repetitive intergenic consensus elements PCR, pulsed-field gel electrophoresis (PFGE) analysis, multiple-locus variable-number tandem-repeats analysis (MLVA), multilocus sequence typing (MLST), multilocus sequence analysis and whole-genome sequencing (WGS) analysis. Molecular subtyping data from sub-Saharan Africa is summarised in Table 1 and the countries involved are highlighted in Figure 1. To date, the most popular and well documented molecular subtyping techniques have included PFGE, MLVA, MLST and WGS analysis. Traditional molecular subtyping methodologies, such as PFGE, MLVA and MLST, have their advantages and disadvantages; these have been well reviewed elsewhere on numerous occasions.^1,2,7,8,9,10,11^ Some molecular subtyping techniques (such as ribotyping, random amplified polymorphic DNA analysis and enterobacterial repetitive intergenic consensus elements PCR) may only allow a local comparison of bacterial strains within a single laboratory, because the workings of the methodology are difficult to standardise between laboratories, and so the data that is produced cannot be truly and accurately compared between laboratories. In contrast, a technique such as PFGE analysis, has been standardised by PulseNet International (http://www.pulsenetinternational.org/) and is successfully used for inter-laboratory comparison of subtyping data and global comparison of bacterial strains.^4^

**TABLE 1:** Summary of data from publications that have described the use of molecular subtyping methodologies to investigate antimicrobial-resistant enteric bacterial pathogens isolated in sub-Saharan Africa.

Year of publication	Type of molecular subtyping method used	Enteric pathogen investigated	Country of isolation of the pathogen	Reason for investigation	Publication reference
1991	Plasmid profiling	*Salmonella* Enteritidis	Unknown countries in Africa	Surveillance	16
1996	PFGE, plasmid profiling	*Shigella dysenteriae*	Kenya	Outbreak	6
1996	PFGE	*Shigella dysenteriae*	Central African Republic	Outbreak	75
1996	Ribotyping	*Vibrio cholerae*	Guinea-Bissau	Outbreak	43
1997	Ribotyping	*Shigella dysenteriae*	South Africa	Outbreak	74
1997	ERIC-PCR	*Vibrio cholerae*	Angola	Outbreak	44
1998	Ribotyping	*Vibrio cholerae*	Senegal	Outbreak	45
1999	PFGE	*Salmonella* Typhimurium	Kenya	Surveillance	17
2001	Ribotyping	*Vibrio cholerae*	Mozambique, South Africa	Outbreak	46
2001	ERIC-PCR	*Vibrio cholerae*	Mozambique, South Africa	Outbreak	47
2003	PFGE	DEC	Nigeria	Outbreak	79
2004	PFGE	*Salmonella* Enteritidis	Tanzania	Outbreak	28
2004	PFGE	*Salmonella* Typhi	Kenya	Outbreak	32
2006	PFGE	*Salmonella* Isangi	South Africa	Outbreak	30
2006	Ribotyping, RAPD analysis	*Vibrio cholerae*	Kenya	Outbreak	52
2007	PFGE	*Vibrio cholerae*	South Africa	Outbreak	56
2008	RAPD analysis	*Vibrio cholerae*	Ghana	Outbreak	50
2008	PFGE	*Vibrio cholerae*	Namibia	Outbreak	57
2009	MLST	*Salmonella* Typhimurium	Kenya, Malawi	Surveillance	20
2009	PFGE	*Shigella boydii*	South Africa	Outbreak	76
2009	PFGE, Ribotyping	*Vibrio cholerae*	Cameroon	Outbreak	53
2009	Ribotyping, RAPD analysis	*Vibrio cholerae*	Somalia	Outbreak	54
2009	Ribotyping, RAPD analysis	*Vibrio cholerae*	Ethiopia	Outbreak	55
2010	PFGE	*Salmonella* Enteritidis	Nigeria	Outbreak	27
2010	PFGE	*Salmonella* Typhi	South Africa	Surveillance	33
2010	PFGE	*Salmonella* Typhi	South Africa	Outbreak	37
2010	WGS-SNP analysis	*Salmonella* Typhi	Kenya	Outbreak	39
2011	PFGE	*Salmonella* Enteritidis	South Africa	Outbreak	26
2011	PFGE	*Salmonella* Typhi	South Africa	Outbreak	4
2011	PFGE	*Vibrio cholerae*	South Africa	Outbreak	48
2011	PFGE, WGS-SNP analysis	*Vibrio cholerae*	Cameroon, South Africa	Research	62
2011	MLSA	*Vibrio cholerae*	Ghana	Outbreak	63
2011	WGS-SNP analysis	*Vibrio cholerae*	Several countries	Research	64
2012	PFGE	DEC	South Africa	Surveillance	80
2012	PFGE	*Salmonella* Typhi	Malawi, Mozambique	Outbreak	34
2012	PFGE	*Salmonella* Typhi	Uganda	Outbreak	35
2012	WGS-SNP analysis	*Salmonella* Typhimurium	Malawi	Surveillance	24
2013	PFGE, MLST, WGS-SNP analysis	*Salmonella* Typhimurium	DRC, Nigeria	Surveillance	22
2013	PFGE	*Vibrio cholerae*	South Africa	Outbreak	58
2013	WGS-SNP analysis	*Vibrio cholerae*	Kenya	Outbreak	65
2013	PFGE, MLSA	*Vibrio cholerae*	Nigeria	Outbreak	68
2014	PFGE	*Salmonella* Typhi	Uganda	Outbreak	36
2014	PFGE, MLVA, MLST	*Salmonella* Typhimurium	South Africa	Outbreak	31
2014	PFGE	*Vibrio cholerae*	Kenya	Outbreak	69
2014	PFGE	*Vibrio cholerae*	Sierra Leone	Outbreak	70
2015	WGS-SNP analysis	*Salmonella* Typhi	Several countries	Research	5
2015	WGS-SNP analysis	*Salmonella* Typhi	Malawi	Surveillance	40
2015	WGS-SNP analysis	*Salmonella* Typhi	Zambia	Outbreak	41
2015	WGS-SNP analysis	*Salmonella* Typhimurium	Kenya	Outbreak	21
2015	MLST	*Salmonella* Typhimurium	South Africa	Surveillance	23
2015	WGS-SNP analysis	*Shigella flexneri*	Several countries	Research	77
2015	PFGE	*Vibrio cholerae*	DRC, Guinea, Togo, Côte d’Ivoire, Mozambique	Outbreak	71
2015	MLVA	*Vibrio cholerae*	DRC, Zambia, Guinea, Togo	Outbreak	72
2016	WGS-SNP analysis	*Salmonella* Enteritidis	Several countries	Research	25
2016	WGS-SNP analysis	*Salmonella* Typhi	Nigeria	Surveillance	42
2016	WGS-SNP analysis	*Shigella dysenteriae*	Several countries	Research	78
2016	WGS-SNP analysis	*Vibrio cholerae*	Cameroon	Outbreak	67
2016	PFGE, MLVA, MLST	*Vibrio cholerae*	Ghana	Outbreak	73
2017	MLVA	*Salmonella* Enteritidis	South Africa	Outbreak	29
2017	MLVA	*Salmonella* Typhi	South Africa	Surveillance	38
2017	MLVA, WGS-SNP analysis	*Vibrio cholerae*	Tanzania	Outbreak	66

DEC, diarrhoeagenic *E. coli*; ERIC-PCR, enterobacterial repetitive intergenic consensus polymerase chain reaction; MLSA, multilocus sequence analysis; MLST, multilocus sequence typing, MLVA, multiple-locus variable-number tandem-repeats analysis; PFGE, pulsed-field gel electrophoresis; RAPD, random amplified polymorphic DNA; WGS-SNP, whole-genome sequencing single nucleotide polymorphism.

Newer approaches to molecular subtyping involve WGS analysis.^12,13,14^ Analysis of WGS data for molecular epidemiological purposes can include multiple approaches; however, the more popular methods are whole-genome MLST and single nucleotide polymorphisms (SNP) analysis.^15^ WGS data is electronically portable and can easily be shared between laboratories, allowing for a global inter-laboratory comparison of bacterial strains. Current challenges surrounding WGS include how to standardise the quality of WGS data generated and how to standardise the analysis of WGS data, in order to allow for a successful inter-laboratory comparison of analysed WGS data. PulseNet International has taken on this challenge and published their vision for implementation of WGS for global foodborne disease surveillance and global analysis of enteric pathogens.^15^

The aim of the present manuscript is to review all publications that have described the use of molecular subtyping methodologies to investigate outbreaks due to multidrug-resistant enteric bacterial pathogens in sub-Saharan Africa. The review focuses on enteric pathogens belonging to the genus and species of *Salmonella, Vibrio cholerae, Shigella* and *Escherichia coli*, because most molecular epidemiological investigations have been described for these microorganisms. The aim of this review is to inform the readers about the current status of molecular subtyping capabilities and activities in sub-Saharan Africa and finally suggest that the way forward for molecular subtyping in African laboratories is the implementation of WGS analysis as soon as possible.

A PubMed database literature search (English language only) was performed using the search strings: ‘Africa outbreak MDR’, ‘Africa outbreak multi’, ‘Africa outbreak multidrug’, ‘Africa outbreak multi drug’, ‘Africa outbreak resistance’, ‘Africa outbreak resistant’, ‘Africa outbreak drug’, ‘Africa outbreak antibiotic’, ‘Africa outbreak antimicrobial’. These search strings were used in combination with genus and species names of the organisms listed above. All results were included in the review.

## Nontyphoidal *Salmonella*

For nontyphoidal *Salmonella* (NTS), molecular epidemiological analysis of *Salmonella* Typhimurium and *Salmonella* Enteritidis are most commonly described in sub-Saharan Africa. One of the first reports using molecular subtyping techniques to investigate enteric bacterial pathogens isolated in sub-Saharan Africa was a 1991 study that used plasmid profiling (following restriction endonuclease digestion) to distinguish African isolates of *Salmonella* Enteritidis from United States (US) isolates.^16^ Molecular subtyping of NTS then progressed to PFGE analysis of MDR *Salmonella* Typhimurium isolated in Kenya, where 64 isolates were grouped into eight PFGE clusters and were described as multiclonal.^17^ *Salmonella* Typhimurium accounts for a significant proportion of reported cases of NTS-associated invasive disease in sub-Saharan Africa. NTS-associated invasive disease has developed to become a leading public health challenge in sub-Saharan Africa.^18,19^ A predominant type of invasive *Salmonella* Typhimurium in sub-Saharan Africa is a MDR strain that has been designated ST313 (based on its MLST profile). Clonal spread of this MDR ST313 strain was first reported from Kenya and Malawi in 2009, with the strain showing resistance to ampicillin, chloramphenicol, sulphamethoxazole, trimethoprim, streptomycin and kanamycin.^20^ In Kenya, a more recent analysis of *Salmonella* Typhimurium outbreak isolates, using WGS-SNP analysis, identified a single clade of MDR ST313 strains showing resistance to ampicillin, ceftriaxone, chloramphenicol, sulphamethoxazole and trimethoprim; extended-spectrum beta lactamase (ESBL) genes *bla*_CTX-M-15_, *bla*_TEM-1_ and *bla*_OXA-1_ were also harboured by the isolates.^21^ The MDR ST313 has further been reported from Malawi, Nigeria, Democratic Republic of the Congo and South Africa.^22,23,24^ Leekitcharoenphon and coworkers^22^ used MLST, PFGE and WGS-SNP analysis to describe a close relationship between MDR ST313 strains from Nigeria and Democratic Republic of the Congo; strains were isolated from invasive (blood) and non-invasive (stool) specimens; all isolates harboured *bla*_TEM1b_, *catA1, strA/B, sul1*, and *dfrA1* genes coding for resistance to various classes of antimicrobial agents. Keddy and coworkers^23^ used MLST to investigate *Salmonella* Typhimurium isolates from South Africa and reported that NTS meningitis in South Africa was highly associated with the MDR ST313 strain.

Besides *Salmonella* Typhimurium, *Salmonella* Enteritidis is also a major player with regard to NTS-associated invasive disease in sub-Saharan Africa. Feasey and coworkers^25^ used WGS-SNP analysis to investigate 675 *Salmonella* Enteritidis isolates from 45 countries (including 28 African countries), to describe the existence of one global epidemic clade and two ‘African’ clades, of which the African clades are geographically confined to specific regions of sub-Saharan Africa. Both African clades show MDR, with an enlarged MDR virulence plasmid. Both African clades also show patterns of genomic degradation with a similarity to those shown by other host-restricted invasive *Salmonella* serotypes described in Africa: patterns of genomic degradation similar to the African *Salmonella* Typhimurium ST313 clade.

Pulsed-field gel electrophoresis analysis is commonly used to investigate outbreaks of *Salmonella* Enteritidis.^26,27,28^ Niehaus and coworkers^26^ reported on a *Salmonella* Enteritidis foodborne outbreak in South Africa, where human isolates and a food isolate were shown to have an indistinguishable PFGE pattern. *Salmonella* Enteritidis investigations have also included nosocomial outbreaks; Vaagland and coworkers^28^ investigated a nosocomial outbreak of neonatal *Salmonella* Enteritidis in Tanzania, with PFGE analysis of MDR (ampicillin, chloramphenicol and cefuroxime) isolates suggesting a clonal outbreak. More recently, MLVA has proven a suitable method to investigate the molecular epidemiology of *Salmonella* Enteritidis in South Africa; MLVA was used to investigate multiple foodborne outbreaks over the period 2013–2015; MLVA was able to cluster outbreak isolates according to distinct MLVA profiles.^29^ Nosocomial outbreaks have also been investigated in South Africa, where *Salmonella* Isangi and *Salmonella* Typhimurium have been involved.^30,31^ Wadula and coworkers^30^ used PFGE analysis to show clonality among ESBL-producing *Salmonella* Isangi in paediatric hospital wards. Smith and coworkers^31^ used PFGE analysis, MLVA and MLST to investigate an ESBL-producing *Salmonella* Typhimurium outbreak in a paediatric hospital ward; outbreak isolates showed indistinguishable molecular subtyping profiles including an MLST subtype of ST34, and showed resistance to ampicillin, ceftriaxone, trimethoprim, sulphamethoxazole, chloramphenicol and tetracycline.

## Typhoidal *Salmonella*

In 2004, Kariuki and coworkers^32^ documented one of the first reports using molecular subtyping techniques to characterise MDR *Salmonella* Typhi isolated in Africa. They used PFGE analysis to investigate 102 Kenyan outbreak isolates of *Salmonella* Typhi and identified two distinct subtypes among the infecting isolates; 82% of the isolates were MDR with resistance to ampicillin, chloramphenicol, tetracycline, streptomycin and cotrimoxazole. When PFGE analysis is standardised, as is the case with PulseNet methodology employed by participating PulseNet International laboratories, then inter-laboratory comparison of PFGE patterns can occur, to allow for a global investigation of outbreak isolates and tracking of emerging strains. Such was the case reported by Smith and coworkers,^4^ who used PFGE analysis to investigate an outbreak of *Salmonella* Typhi associated with a restaurant in South Africa; the source of the infecting strain was a restaurant worker who was tracked to Australia where a *Salmonella* Typhi isolate was recovered from the individual; the isolate shared an indistinguishable PFGE pattern as compared to the pattern of the outbreak strain. Using the same scenario as described above (comparison of PFGE patterns within the PulseNet International laboratory network), Keddy and coworkers^33^ investigated a MDR (including resistance to fluoroquinolones) *Salmonella* Typhi isolate recovered from a South African patient who had interacted with a person who had recently travelled to Bangladesh; the PFGE pattern of the isolate had never previously been seen within the South African PFGE database, but the PFGE pattern was typical of that seen of isolates from the Indian subcontinent, evidence to support the view that the isolate originated in Bangladesh. Global tracking of *Salmonella* Typhi through inter-laboratory comparison of molecular subtyping data is particularly important in establishing the source (and spread) of MDR (and virulent) strains of *Salmonella* Typhi. An example of such a strain is *Salmonella* Typhi H58, a highly clonal MDR haplotype of *Salmonella* introduced from Asia into Africa and now currently spreading through many sub-Saharan African countries.^5^

Further investigation of *Salmonella* Typhi outbreaks using PFGE analysis have been reported from the Malawi-Mozambique border,^34^ in Uganda^35,36^ and in South Africa.^37^ In Uganda, Walters and coworkers^36^ investigated a prolonged waterborne outbreak in two neighbouring districts; they used PFGE analysis to document the clonal spread of MDR *Salmonella* Typhi from the Kasese district to the Bundibugyo district. Analysis of selected isolates showed MDR to ampicillin, chloramphenicol, cotrimoxazole, streptomycin and tetracycline. In South Africa, Keddy and coworkers^37^ investigated a waterborne outbreak in the Delmas area of South Africa in 2005; they used PFGE analysis and MLVA to show high relatedness among outbreak isolates. These outbreak isolates from 2005 were also shown to be highly related to isolates associated with a similar waterborne outbreak in the same area in 1993. Besides PFGE analysis, MLVA is often described as a useful molecular subtyping method for discrimination of strains belonging to the *Salmonella* species. Tau and coworkers^38^ described the development and evaluation of a MLVA assay for molecular subtyping of *Salmonella* Typhi in sub-Saharan Africa. They evaluated the MLVA assay on a panel of African isolates and showed that it had higher discriminatory power as compared to PFGE; the MLVA assay was able to differentiate outbreak isolates from sporadic isolates.

Whole-genome sequencing – single nucleotide polymor-phisms analysis has been increasingly used to investigate the molecular epidemiology of *Salmonella* Typhi in sub-Saharan Africa; a common theme has been the description of the MDR *Salmonella* Typhi H58 strain.^5,39^ Wong and coworkers^5^ used WGS-SNP analysis to investigate 1832 *Salmonella* Typhi isolates from 63 global countries, to identify one major MDR lineage (named H58) that has arisen and spread across Asia and Africa over the past 30 years. Multiple H58 transfers have occurred, moving from Asia into Africa. An MDR H58 epidemic has been described in sub-Saharan Africa where H58 lineages are displacing antimicrobial-susceptible strains. MDR H58 lineages are associated with resistance to ampicillin, trimethoprim, sulphonamides, chloramphenicol, streptomycin and tetracycline, including reduced susceptibility to fluoroquinolones.^5,39^ This MDR H58 lineage has now been described in the eastern and southern regions of sub-Saharan Africa including Kenya, Tanzania, Malawi and South Africa.^5,38,39,40^ Hendriksen and coworkers^41^ also used WGS-SNP analysis to investigate an outbreak of *Salmonella* Typhi in Zambia; most isolates showed resistance to ampicillin, chloramphenicol, streptomycin, sulphamethoxazole and trimethoprim, while some isolates also had reduced susceptibility to fluoroquinolones. Isolates belonged to a new variant of the H58 haplotype, namely haplotype H58B. Interestingly, investigation of MDR *Salmonella* Typhi from Nigeria -western Africa, using WGS-SNP analysis, has not detected the H58 lineage; instead the majority of isolates belonged to a different lineage -H56 lineage, which carries genes coding for resistance to ampicillin, tetracycline, chloramphenicol and sulphamethoxazole (*bla*_TEM1_, *catA1, tetB, dfrA1, sul1*). This H56 lineage is relatively common across Africa, predominantly in western and central regions of sub-Saharan Africa.^42^

## Vibrio cholerae

Ribotyping and enterobacterial repetitive intergenic consensus elements PCR were among the first molecular subtyping techniques used to characterise *V. cholerae* O1 in Africa.^43,44^ In 1996, Dalsgaard and coworkers^43^ documented the first molecular epidemiological investigations of MDR *V. cholerae* O1 in Africa. They used ribotyping to investigate outbreak isolates in Guinea-Bissau; strains isolated in 1994–1995 showed a ribotype pattern which was different fro strains isolated in 1987, suggesting that the 1994–1995 outbreak was as a result of the introduction of a novel strain into the country. Later, ribotyping analysis of *V. cholerae* O1 outbreak isolates from Senegal showed the same ribotype pattern as the 1994–1995 Guinea-Bissau outbreak strain, suggesting that Senegal acquired their outbreak strain from Guinea-Bissau.^45^ In 2001, Dalsgaard and coworkers^46^ reported on the clonal relationship (using ribotyping) of cholera isolates from Mozambican migrant workers associated with a 1998 outbreak that occurred in South African provinces bordering Mozambique. MDR *V. cholerae* O1 isolates were associated with the outbreak among the migrant workers and the isolates showed resistance to furazolidone, streptomycin, sulphamethoxazole, trimethoprim and tetracycline; isolates also showed the presence of class 1 integrons and the SXT element. Another analysis of Mozambican MDR isolates associated with the 1998 Mozambique/South Africa cholera outbreak confirmed the presence of the SXT element among isolates, with enterobacterial repetitive intergenic consensus elements PCR dividing isolates into two different molecular subtypes.^47^ Mobile genetic elements are mostly responsible for the molecular basis of MDR *V. cholerae* O1 in sub-Saharan Africa. Mobile genetic elements include transposable elements (SXT elements), integrons and conjugative plasmids. The SXT element is a self-transmissible element that integrates into the chromosome and carries genes encoding resistance to several antimicrobial agents, including chloramphenicol, sulfamethoxazole, streptomycin, trimethoprim and furazolidone. The SXT element has been reported from numerous countries in sub-Saharan Africa.^46,48,49,50,51^ In 2006, Scrascia and coworkers^52^ investigated *V. cholerae* O1 isolates from multiple outbreaks that occurred in Kenya during 1998 and 1999. Most isolates showed an identical ribotype profile and similar random amplified polymorphic DNA analysis profile suggesting a clonal origin for the outbreaks; isolates were resistant to chloramphenicol, spectinomycin, streptomycin, sulphamethoxazole and trimethoprim. Over the period 2008–2009, published data from Ghana, Cameroon, Ethiopia and Somalia reported similar trends (as described above) for MDR *V. cholerae* O1 isolates following analysis using ribotyping and random amplified polymorphic DNA analysis - that of each country reporting a clonal origin for outbreak isolates within their respective country.^50,53,54,55^

From 2007 onwards, trends in molecular subtyping of *V. cholerae*, started shifting to an increased use of PFGE analysis. In 2007, Keddy and coworkers^56^ used PFGE analysis to compare the relatedness of South African *V. cholerae* O1 isolates from a 2001–2002 epidemic to that of a 1980–1987 epidemic; PFGE analysis showed that isolates from the 1980–1987 epidemic were distinctly different to isolates from the 2001–2002 outbreak. In 2008, Smith and coworkers^57^ reported on the first cholera outbreak in Namibia over the period 2006–2007; MDR *V. cholerae* O1 isolates showed an indistinguishable PFGE pattern and showed resistance to streptomycin, sulphamethoxazole and trimethoprim. In 2011, Ismail and coworkers^48^ investigated an outbreak of ESBL-producing MDR *V. cholerae* O1 in South Africa which occurred over the period May to July 2008; PFGE analysis showed a clonal relationship among isolates, with isolates showing resistance to ampicillin, cotrimoxazole, chloramphenicol, nalidixic acid, tetracycline, kanamycin and streptomycin. The molecular basis of antimicrobial resistance was explained by proving the presence of the SXT element and the *bla*_TEM_ gene encoding TEM-63 β-lactamase. Later in 2008, a *V. cholerae* O1 outbreak in Zimbabwe spilled over into South Africa, which triggered a very large outbreak in South Africa over the period November 2008 to April 2009. MDR outbreak isolates showed similar PFGE patterns: isolates showed the presence of the SXT element encoding multidrug resistance; some isolates also showed ESBL activity due to the presence of the *bla*_TEM_ gene encoding TEM-63 β-lactamase.^58^ In October 2010, the Haiti cholera outbreak started, with involvement of MDR *V. cholerae* O1.^59,60^ PFGE analysis of Haiti outbreak strains isolated over the period October 2010 to February 2011 showed that a single PFGE profile predominated. This predominant profile was also shown in MDR strains from Cameroon, South Africa, India, Nepal, Pakistan, Afghanistan and Oman^61^; this global comparison of *V. cholerae* O1 PFGE patterns was facilitated via PulseNet International laboratories. Further investigation with WGS-SNP analysis showed that the Haiti outbreak strain was a hybrid-type of El Tor strain encoding a classical-type of cholera toxin and that the outbreak strain was most closely related to strains originating from India and Cameroon.^62^

The Haiti cholera outbreak identified the need for higher resolution molecular subtyping methodologies for comparison of *V. cholerae* O1 isolates; this sparked the increased use of nucleotide sequencing approaches including WGS, to generate nucleotide sequence data for comparison of isolates. In 2011, Thompson and coworkers^63^ characterised *V. cholerae* O1 outbreak isolates from Ghana using multilocus sequence analysis of housekeeping genes and identified two major clusters in Ghana. In 2011, Mutreja and coworkers^64^ provided evidence for multiple waves of global transmission (from the Bay of Bengal) within the seventh cholera pandemic, which included multiple waves of transmission into sub-Saharan Africa; these data were made possible by analysis of WGS data (WGS-SNP analysis approach) from a global collection of *V. cholerae* O1 isolates, including African outbreak isolates. In 2013, Kiiru and coworkers,^65^ reported a WGS-SNP analysis on Kenyan *V. cholerae* isolates which included environmental isolates (serogroups O1 and non-O1) and *V. cholerae* O1 clinical isolates associated with outbreaks; they found that a clade comprising clinical isolates and some environmental isolates mapped back onto wave three of the monophyletic seventh cholera pandemic phylogeny, while some other environmental isolates were phylogenetically very different from the monophyletic seventh pandemic lineage of *V. cholerae* O1. The continued presence of MDR associated STX elements were documented on the genomes of MDR clinical and MDR environmental isolates. Recent isolates of *V. cholerae* O1 from Tanzania have also been found to map back onto wave three of the monophyletic seventh cholera pandemic phylogeny, as determined by WGS-SNP analysis.^66^ Interestingly, WGS-SNP analysis of MDR *V. cholerae* O1 outbreak isolates recently sourced from Cameroon has found that these Cameroonian isolates do not map onto the phylogeny of isolates from other African countries such as Kenya, Tanzania, Zambia, Zimbabwe and Mozambique; instead, Cameroonian isolates are of their own distinct clonal cluster (clade), forming part of an isolated reservoir of MDR *V. cholerae* O1 in the Lake Chad basin.^67^

Although WGS approaches for comparison of *V. cholerae* isolates have gained a stronghold in sub-Saharan Africa, the traditional molecular subtyping methodologies of PFGE, MLVA and MLST are still commonly used to investigate the molecular epidemiology of *V. cholerae* in sub-Saharan Africa, as these technologies still remain more accessible and more affordable for most laboratories^66,68,69,70,71,72,73^. For these recent reports, the central themes remain the same as previous studies. The major conclusions for these studies are summarised as follows. The most commonly reported cholera-causing organism is characterised as *V. cholerae* O1 biotype El Tor, the so-called ‘atypical’ El Tor variant housing genetic determinants coding for a ‘classical-type’ of cholera toxin. The majority of *V. cholerae* O1 isolates show the presence of SXT elements encoding a large part of the MDR phenotype. The majority of isolates show MDR. A common profile includes resistance to ampicillin, chloramphenicol, sulphamethoxazole, trimethoprim, spectinomycin and streptomycin, including reduced susceptibility to ciprofloxacin. Molecular subtyping of *V. cholerae* O1 isolates shows examples of geographical clustering, as already described above in the mention of Cameroonian isolates characteristic of the Lake Chad basin region; Moore and coworkers^72^ found that isolates from western Africa (Togo and Guinea) form a closely related group which is separated from a distinctive cluster associated with the African Great Lakes region (Democratic Republic of the Congo and Zambia). Smith and coworkers^71^ found that a Mozambican cluster of isolates was distinctly different (including a different antimicrobial resistance profile) as compared to isolates from western Africa (Togo, Guinea and Côte d’Ivoire) and central Africa (Democratic Republic of the Congo).

## *Shigella* species

*Shigella dysenteriae* was the first *Shigella* species in sub-Saharan Africa that was characterised by molecular subtyping techniques; these details were first documented in the years 1996 and 1997.^6,74^ In 1996, Kariuki and coworkers^6^ documented an outbreak of dysentery due to MDR *S. dysenteriae* serotype 1 in Kenya: all 22 outbreak isolates were resistant to ampicillin, chloramphenicol, sulphamethoxazole, trimethoprim, streptomycin and tetracycline; PFGE and plasmid profiling showed clonality among the isolates. In 1997, Pillay and coworkers^74^ documented a nosocomial outbreak of dysentery caused by MDR *S. dysenteriae* serotype 1 which transpired in a psychiatric institution in South Africa. Isolates were resistant to ampicillin, chloramphenicol, tetracycline and cotrimoxazole; ribotyping showed clonality among the isolates. Later in 1996, a report documented how PFGE analysis was used to investigate MDR *S. dysenteriae* serotype 1 isolates associated with two separate dysentery outbreaks in the Central African Republic; PFGE analysis provided evidence to show that each outbreak was associated with a different clone.^75^ In 2009, Smith and coworkers^76^ reported a cluster of 29 watery diarrhoea cases associated with MDR *Shigella boydii* serotype 2 isolates in South Africa. Isolates were resistant to ampicillin, trimethoprim, sulphamethoxazole and streptomycin; PFGE analysis showed clonality among the isolates.

More recent molecular epidemiological investigations of *Shigella* species in sub-Saharan Africa has involved WGS-SNP analysis of isolates. Connor and coworkers^77^ used WGS-SNP analysis to investigate 351 global (including Africa) isolates of *Shigella flexneri*, to show that *S. flexneri* is composed of seven distinct phylogenetic groups. Each phylogenetic group contains geographically restricted sub-lineages that seem to have constantly inhabited areas for several decades; there is limited evidence of intercontinental transmission. Antimicrobial resistance determinants have been acquired and maintained locally on several episodes – to summarise, *S. flexneri* from sub-Saharan Africa cluster together and form a distinct lineage as compared to lineages from other parts of the world. Njamkepo and coworkers^78^ used WGS-SNP analysis to investigate 331 global (including Africa) isolates of *S. dysenteriae* serotype 1, to show that *S. dysenteriae* serotype 1 is composed of four distinct lineages. Lineage IV contains most of the recent (last few decades) isolates from Africa and the Indian subcontinent; lineage IV has been transmitted in several waves to Africa; most recent (since 1990) outbreaks in Africa have been caused by this lineage IV; compared to other lineages, lineage IV show antimicrobial resistance including MDR.

## Escherichia coli

Surprisingly very little published data exist concerning molecular epidemiological investigation of outbreaks in sub-Saharan Africa caused by *E. coli* enteric pathogens (diarrhoeagenic *E. coli*). Diarrhoeagenic *E. coli* includes the following six main categories: shiga-toxin producing *E. coli* (which includes the enterohaemorrhagic *E. coli*), enteropathogenic *E. coli*, enteroinvasive *E. coli*, enterotoxigenic *E. coli*, enteroaggregative *E. coli* and diffusely adherent *E. coli*. In 2003, Okeke and coworkers^79^ reported on the etiology of acute diarrhoea in adults in Nigeria: shiga-toxin producing *E. coli* and enteroaggregative *E. coli* were significantly associated with the diarrhoea. PFGE analysis of shiga-toxin producing *E. coli* isolates provided evidence to suggest that an outbreak had occurred. In 2012, Tau and coworkers^80^ reported on the characterisation of *E. coli* O104 isolates from South Africa for the years 2004–2011. This study was undertaken in response to the 2011 outbreak of bloody diarrhoea and haemolytic uraemic syndrome which occurred in Germany, caused by an enteroaggregative shiga-toxin producing *E. coli* O104:H4^81^. The South African investigation found two antimicrobial-susceptible enteropathogenic *E. coli* O104:non-H4 isolates and five enteroaggregative *E. coli* O104:H4 isolates (showing resistance to ampicillin, sulphamethoxazole and trimethoprim). Pulsed-field gel electrophoresis analysis showed that these South African isolates were unrelated to the Germany outbreak strain.

### Conclusion

Whole-genome sequencing is undoubtedly the way forward for investigation of enteric pathogens in diagnostic and public health laboratories. Numerous publications have analysed data sets to compare results obtained using old-fashioned molecular subtyping methodologies versus results obtained using newer WGS analysis methodologies.^14,82,83,84^ Conclusions from these studies are unanimous and convincing, in that WGS trumps all other methodologies. Old-fashioned methodologies offer limited genetic resolution and limited discriminatory ability and there can sometimes be a lack of accuracy; all these hinder epidemiologic investigations. For example, data from old-fashioned methodologies can incorrectly conclude that unrelated isolates are indistinguishable, resulting in false alerts and unnecessary investigations. Whole-genome sequencing provides a supreme unparalleled discriminatory ability, can reflect phylogeny and can provide an evolutionary context. In addition, WGS can provide valuable information related to genetic determinants conferring antimicrobial resistance. Numerous publications have reported how analysis of WGS data can be used to infer antimicrobial resistance in bacterial pathogens;^85,86,87,88,89^ these studies have reported high concordance between WGS resistance predictions and conventional phenotypic antimicrobial susceptibility testing methodologies. Even so, conventional phenotypic susceptibility testing methodologies still have their place in the laboratory and must continue to be used for the foreseeable future. For now, WGS antimicrobial resistance prediction is best used as an adjunct to conventional phenotypic susceptibility testing.

In summary, public health laboratories in Africa need to urgently reconsider and adapt their molecular surveillance methodologies to include WGS. It is foreseen that WGS analysis will eventually replace all traditional methods currently used in the microbiology laboratory, both traditional phenotypic methods and traditional genotypic methods including molecular subtyping methods such as PFGE, MLVA and MLST.^90,91,92,93^ Within the next year, the PulseNet USA programme is planning to have WGS (and analysis of WGS data) fully implemented as its primary method for molecular epidemiological investigation of enteric pathogens.^94^ The next challenge is how to effectively implement WGS analysis globally in public health laboratories, how to standardise the quality of WGS data generated and how to standardise the analysis of WGS data, all in order to allow for a successful inter-laboratory comparison of analysed WGS data. PulseNet International has taken this challenge and published their vision for implementation of WGS for global foodborne disease surveillance and global analysis of enteric pathogens.^15^ PulseNet Africa (a regional network of PulseNet International) believes and supports this vision for implementation of WGS for global analysis of enteric pathogens.
